# Deep Bite Correction with the Invisalign System: A Case-Series Study

**DOI:** 10.1055/s-0045-1808255

**Published:** 2025-06-24

**Authors:** Trey Spillers, David Alexandre Cruz Walma, Jerry Don Spillers, Chung How Kau, Terpsithea Christou

**Affiliations:** 1 Department of Orthodontics, School of Dentistry, University of Alabama at Birmingham, Birmingham, Alabama, United States; 2 Department of Orthodontics, Harvard School of Dental Medicine, Boston, Massachusetts, United States

**Keywords:** deep bite, Invisalign, clear aligner therapy, anterior tooth intrusion, posterior tooth extrusion

## Abstract

**Objective:**

This article assesses the dental and skeletal changes resulting from correction of a deep bite using Invisalign clear aligner therapy (Align Technology, Santa Clara, California, United States).

**Materials and Methods:**

This is a retrospective case series study that analyzed pre- and posttreatment cephalograms for 37 adult deep bite patients. Each patient was treated by one experienced clinician using the same treatment protocol. Ten linear and 9 angular variables were evaluated pre- and posttreatment. The Wilcoxon analysis was used to determine if there was a significant difference in the 19 variables pre- and posttreatment.

**Results:**

Statistically significant differences ( *p* < 0.05) were observed for 14 of 19 variables. These included a decrease in overjet (–1.0 mm), decrease in overbite (–4.1 mm), extrusion of the upper first molar (+0.5 mm), intrusion of the upper incisor (–0.6 mm), extrusion of the lower second molar (+1.0 mm), lower first molar (+1.2 mm), lower second premolar (+1.70 mm), and lower first premolar (+1.7 mm), intrusion of the lower incisor (–1.8 mm), proclination of lower incisor (+4.1 degrees), and an increase in sella-nasion- mandibular plane (+0.6 mm). A decrease in anterior occlusal plane was also significant.

**Conclusion:**

Invisalign is an effective treatment modality for correcting dental deep bites through posterior extrusion and anterior intrusion. Minimal skeletal changes are seen when correcting deep bites with Invisalign. Results of this study enhance our understanding of the dental and skeletal changes that can be expected when attempting to correct deep bites with clear aligner therapy.

## Introduction

The field of orthodontics experienced a transformation with the introduction of the Invisalign System by Align Technology in 1998. 1 Since its inception, orthodontists have attempted to understand the limitations and capabilities of clear aligner therapy (CAT) in addressing various dental malocclusions. 2 3 Historically, the idea of employing plastic materials for repositioning teeth is not novel. Indeed, this concept was introduced as early as the mid-20th century. 4 The contemporary Invisalign System has leveraged digital technology through incorporating patient-specific three-dimensional (3D) modeling, allowing clinicians to visualize and manipulate treatment plans using the “Clincheck interface.” 5 Upon its introduction, Invisalign was mainly marketed to adults presenting with minor dental misalignments, emphasizing its esthetic edge over conventional fixed appliances. 6 Notably, its transparent nature, user comfort, and facilitation of oral hygiene were some of its key selling points. 5 Initial studies suggested clear aligners were only to be used for “simple malocclusions.” 7 Due to continuous improvements in clear aligner attachments and software alongside expanded clinical experiences, Invisalign's treatment scope has similarly expanded, making it a viable option for more complex malocclusions. 1

Deep bite, characterized by an excessive overlap of the upper front teeth over the lower front teeth in a vertical direction, is a prevalent malocclusion with potential negative implications on both aesthetics and function. 8 The etiology of deep overbite malocclusion is multifactorial, arising from a combination of dental and skeletal factors. 9 10 While a minor deep bite may pose minimal risk for most patients, 11 unaddressed severe or impinging deep bites may traumatize palatal issues. 12 The magnitude of this issue becomes evident when considering prevalence statistics: a study spanning a cohort of 6,768 adolescents (aged 12–17) reported that 38.6% exhibited moderate deep bites (3–5 mm), with 10.3% presenting severe deep bites exceeding 6 mm. 13

Correcting severe deep bites with CAT remains a challenging orthodontic endeavor and a topic of interest among orthodontists. With more and more patients wishing to receive CAT over standard fixed appliances, the ability to reproducibly correct severe deep bites with CAT would be of significant benefit to the orthodontic community. The aim of this study was to evaluate the ability to use CAT with Invisalign to correct severe deep bite malocclusions. Results of this study will help shed light on the dental and skeletal changes that can be expected when attempting to correct severe deep bites with CAT.

## Materials and Methods

The study was conceived as a retrospective case series. The Institutional Review Board (IRB) of the University of Alabama, Birmingham, United States reviewed and subsequently approved the study's protocol (Protocol No. IRB#300010568). The study sample was comprised of adults who had completed their growth phase and were sequentially treated using Invisalign by a single skilled Invisalign provider. This provider has treated more than 10,000 Invisalign cases and is among the top 1% of Invisalign practitioners in the United States. Data from patients was sourced from two private clinics located in Warner Robins, United States and Macon GA, United States. Utilizing the OrthoTrac (Carestream Dental, Georgia, United States) practice management software, a comprehensive report was generated using the keyword “deep bite” and Invisalign was selected as the treatment modality. All patients included in the study completed treatment between January 1, 2017, and December 31, 2022. From this initial electronic search, a total of 154 cases emerged with pre- and posttreatment two-dimensional (2D) cephalograms. The retrieved cases were reviewed by two investigators (T.S. and T.C.) using preestablished inclusion and exclusion criteria, as detailed in Table 1 . Of the 154 cases initially identified, 117 patients were excluded from the study with a breakdown of reasons for exclusion presented in Table 2 . Thirty-seven patients fulfilled the predefined criteria and were considered for final analysis. The protocol that was used to treat each qualifying case with Invisalign CAT is presented in Table 3 . All qualifying cases were deidentified prior to further analysis.

**Table 1:** Inclusion and exclusion criteria

Inclusion criteria	Exclusion criteria
Male or female 18 years of age or older	Incomplete or poor-quality records
Deep overbite of at least 3 mm prior to treatment	Patients with significant medical histories (syndromes, etc.)
Treated consecutively with Invisalign in both arches	Patients with previously missing teeth other than third molars
Initial and final lateral cephalograms taken within 6 months of initiation and completion of treatment	Patients with implants
Treatment completed between January 1, 2019, and December 31, 2022	Treatment involving orthodontic appliances other than the Invisalign System
All patients treated by the same provider	Treatment involving extractions
	Patients who had restorative work or surgery done prior to final scan

**Table 2:** Overview of reasons for patient exclusion

Exclusion criteria	Number of patients excluded
Under 18 years of age at start of treatment	83
Implants present	7
Missing teeth other than third molars before or during treatment	15
Incomplete or poor-quality records	7
Auxiliaries used other than aligners or elastics	2
Restorative work received during treatment	3
Total excluded	117

**Table 3:** Invisalign deep bite treatment protocol followed in this study

1. To open the bite, extrude U/L 4-5-6s and intrude U/L 3-3
2. Intrude anterior teeth to a 1.5 mm open bite finish with a gradual step down from lower 4-3-2
3. Place precision bite ramps on all upper 2-2 to aid in initial bite opening
4. For the eight bicuspids, ensure there are 4 mm horizontal bar attachments. However, if the software can place large, optimized attachments, then use the optimized attachments
5. Place any optimized attachments as needed, including on the U/L 6's
6. The case should finish with 1.5 mm open bite with heavy posterior contacts. Please do not have a drastic step between the 3s and 4s on the lower arch when intruding the lower anterior teeth

Note: Patients were advised to change their aligners weekly and wear them for 22 hours per day.

The following data was extracted from each case for analysis: (1) lateral cephalograms at two distinct times: pretreatment (IN) and posttreatment (FN), (2) the “Invisalign Treatment Overview Report,” (3) duration of the treatment, (4) age at the initiation of treatment, and (5) patient gender. All information was encrypted for security purposes and stored on a password-protected computer.

**Fig. 1:**
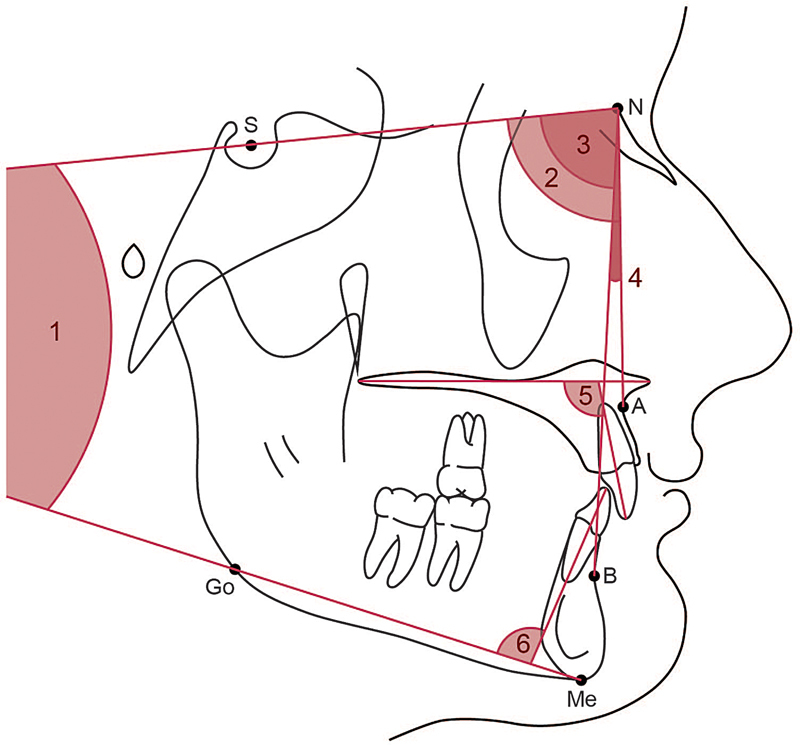
A schematic illustrating the angular measurements evaluated in this study. 1. SN-MP: angle between SN plane and mandibular plane (Go-Me); 2. SNA: angle between SN plane and NA plane; 3. SNB: angle between SN plane and NB plane; 4. ANB: angle between NA plane and NB plane; 5. U1-PP: angle between long axis of the U1 and palatal plane (ANS-PNS); 6, L1-MP: angle between the long axis of the L1 and mandibular plane (Go-Me).

**Fig. 2:**
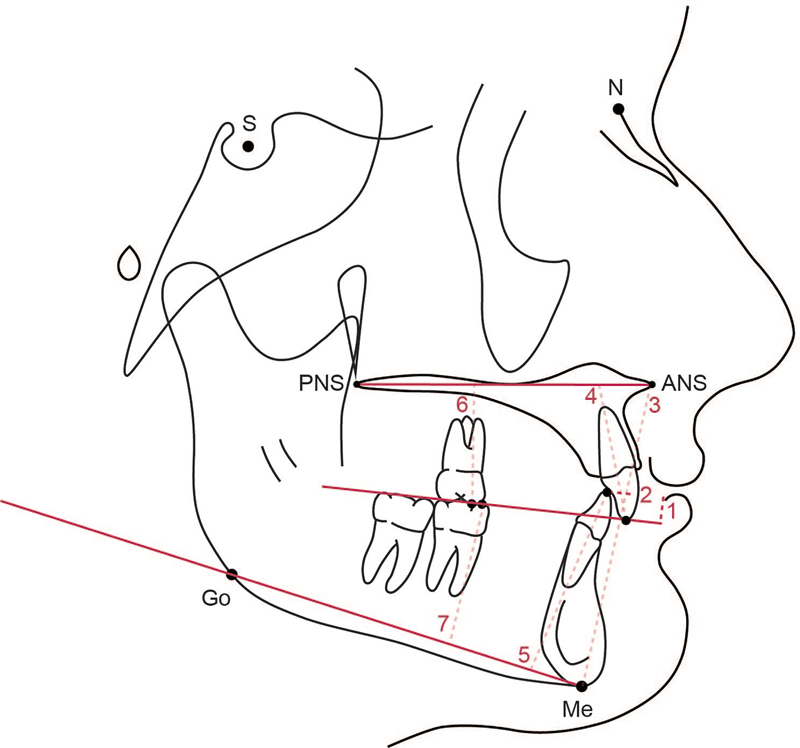
A schematic illustrating the linear measurements evaluated in this study. 1. Overbite: shortest distance from U1 tip and L1 tip perpendicular to the occlusal plane; 2. overjet: distance from the tip of U1 to tip of L1; 3. lower anterior facial height (LAFH): shortest distance from ANS to Me; 4. U1-PP: shortest distance from U1 tip to palatal plane; 5. L1-MP: shortest distance from L1 tip to mandibular plane; 6. U6-PP: shortest distance from the mesiobuccal cusp tip of the U6 to palatal plane; 7. L6-MP: shortest distance from the mesiobuccal cusp tip of the L6 to mandibular plane.

Lateral cephalograms from the 37 qualifying deidentified cases were integrated into the Dolphin Software (Dolphin Imaging, Chatsworth, California, United States). Each lateral cephalogram was digitized and traced using the Dolphin Software. Two investigators (T.S. and T.C.) separately traced each lateral cephalogram to acquire the 19 metrics evaluated in this study. All lateral cephalograms were retraced a second time 2 weeks later to ensure inter- and intraexaminer reliability. The 19 continuous measurements were tested for reliability using the interclass correlation coefficient (ICC). The ICC value correlated from the measurements was 0.94, which suggested excellent reliability. Of the 19 metrics used to assess dental and skeletal growth during the treatment course, 10 were linear and 9 were angular. These metrics are visually represented in Figs. 1 and 2 and their respective definitions are provided in Table 4 . A power test was performed for the sample size of 37 patients and resulted in 99% power to detect a change in the deep bite correction at the 5% significance level. The software used for the power calculation was G*Power version 3.1 9.7. Statistical analyses were performed with Statistical Package for the Social Sciences (SPSS) software (version 28.0.0.0, Armonk, New York, United States). Wilcoxon signed-rank tests were performed to determine whether significant differences existed in pre- and posttreatment variables.

**Table 4:** Definitions of variables assessed over the course of treatment

Variable	Definition
Overjet (mm)	The horizontal millimetric distance from U1 tip to L1 tip
Overbite (mm)	The vertical millimetric distance from U1 tip and L1 tip perpendicular to the occlusal plane
SNA (degrees)	This angle indicates the horizontal position of the maxilla relative to the cranial base. A point is the most anterior measure of the maxillary apical base
SNB (degrees)	This angle indicates the horizontal position of the mandible relative to the cranial base. B point is the most anterior measure of the mandibular apical base
ANB (degrees)	This angle measures the relative position of the maxilla to the mandible. The ANB angle is calculated from the following formula: ANB = SNA – SNB
LAFH (mm)	The millimetric distance between ANS and menton
U6–PP (mm)	The millimetric distance between the mesiobuccal cusp tip of the U6 and the palatal plane (ANS – PNS)
U1–PP (mm)	The millimetric distance between U1 tip and the palatal plane (ANS – PNS)
U1–PP (degrees)	Angle measured by the intersection of the long axis of U1 and palatal plane (ANS – PNS)
L7–MP (mm)	The millimetric distance between the mesiobuccal cusp tip of the L7 and the mandibular plane (Go – Me)
L6–MP (mm)	The millimetric distance between the mesiobuccal cusp tip of the L6 and the mandibular plane (Go – Me)
L5–MP (mm)	The millimetric distance between the buccal cusp tip of the L5 and the mandibular plane (Go – Me)
L4–MP (mm)	The millimetric distance between the buccal cusp tip of the L4 and the mandibular plane (Go – Me)
L1–MP (mm)	The millimetric distance between L1 tip and the mandibular plane (Go – Me)
L1–MP (degrees)	Angle measured by the intersection of the long axis of L1 and mandibular plane (Go – Me)
SN – MP	Angle measured by the intersection of a line connecting sella-nasion (SN) and a line connecting mandibular plane (Go – Me)
AOP	Line connecting the maxillary incisal edge and the averaged cusp tip of the maxillary second premolar
FH – AOP	Angle measured by the intersection of the lines of Frankfort horizontal (FH) and anterior occlusal plane (AOP)
SN – AOP	Angle measured by the intersection of the lines connecting sella- nasion (SN) and the anterior occlusal plane (AOP)
PP – AOP	Angle measured by the intersection of the lines connecting palatal plane (PP) and the anterior occlusal plane (AOP)

Abbreviations: ANB, angle between NA plane and NB plane; AOP, anterior occlusal plane; FH, Frankfort horizontal; LAFH, lower anterior facial height; MP, mandibular plane; PP, palatal plane; SN, sella-nasion; SNA, angle between SN plane and NA plane; SNB, angle between SN plane and NB plane.

## Results

Data from 37 patients was included in the study. Within the sample, 27 patients (73%) were females and 10 (27%) were males. The average pretreatment age of the group was 37 years (standard deviation = 14.8). The youngest patient in the study was 18 years old at the onset of treatment and the oldest was 73 years old. The mean treatment time was 21.1 ± 7.1 months. The shortest treatment duration in the group was 7 months and the longest 34 months. The demographics of the study group and descriptive statistics of the treatments rendered are presented in Table 5 .

**Table 5:** Patient population demographics and study descriptive statistics

Characteristic	Quantity
Total patients	37
Number of males	10
Number of females	27
Age range	18–73 y
Average age	37.32 y
Median age	32 y
Treatment time range	7–34 mo
Average treatment time	21.11 mo
Median treatment time	20 mo

Significant changes over treatment were observed for 14 of 19 variables, including overjet (mean difference = –1.0 mm), overbite (mean difference = –4.1 mm), L7-mandibular plane (MP) (mean difference = +1.0 mm), L6-MP (mean difference = +1.2 mm), L5-MP (mean difference = + 1.7 mm), L4-MP (mean difference = + 1.7), L1-MP (mm) (mean difference = –1.9 mm), L1-MP (degrees) (mean difference = +4.1 degrees), U6-palatal plane (PP) (mean difference = +0.5 mm), U1-PP (mm) (mean difference = –0.7 mm), sella-nasion (SN)-MP (mean difference = +0.5 degrees), SN-anterior occlusal plane (AOP) (mean difference = –1.4 degrees), PP-AOP (mean difference = –1.5), and Frankfort horizontal (FH)-AOP (mean difference = –1.8 degrees). The changes in the angle between SN plane and NA plane (SNA) (mean difference = –0.2 degrees), angle between SN plane and NB plane (SNB) (mean difference = –0.3degrees), angle between NA plane and NB plane (ANB) (mean difference = –0.2 degrees), lower anterior facial height (LAFH) (mean difference = +0.7 mm), and U1-PP ( degrees) (mean difference = +1.2 degrees) were not statistically significant ( Table 6 ).

**Table 6:** Changes in the measured variables observed over the course of treatment

Variable	Initial mean values (SD)	Final mean values (SD)	Final minus initial means values (SD)	*p* -Value
Overjet (mm)	4.0 (1.0)	3.0 (1.0)	–1.0 (0.9)	< 0.001 a
Overbite (mm)	5.2 (1.3)	1.1 (0.7)	–4.1 (1.3)	< 0.001 a
SNA (°)	82.6 (4.1)	82.4 (4.2)	–0.2 (1.4)	0.718
SNB (°)	79.4 (3.7)	79.3 (3.8)	–0.1 (1.0)	0.793
ANB (°)	3.2 (2.6)	3.1 (2.6)	–0.1 (1.2)	0.757
LAFH (mm)	61.6 (6.7)	62.3 (7.0)	+0.7 (2.2)	0.072
U6-PP (mm)	21.2 (2.4)	21.7 (2.6)	+0.5 (1.6)	0.039 a
U1-PP (mm)	27.4 (3.2)	26.8 (3.5)	–0.6 (1.3)	0.003 a
U1-PP (°)	107.4 (8.9)	108.6 (6.9)	+1.2 (7.2)	0.521
L7-MP (mm)	27.8 (3.2)	28.8 (3.3)	+1.0 (1.5)	< 0.001 a
L6-MP (mm)	29.9 (3.5)	31.1 (3.3)	+1.2 (1.6)	< 0.001 a
L5-MP (mm)	30.8 (3.6)	32.5 (3.7)	+1.7 (1.4)	< 0.001 a
L4-MP (mm)	32.3 (3.8)	34.0 (3.8)	+1.7 (1.4)	< 0.001 a
L1-MP (mm)	39.2 (3.9)	37.3 (4.8)	–1.8 (2.6)	< 0.001 a
L1-MP (degree)	92.7 (7.6)	96.8 (6.5)	+4.1 (5.7)	< 0.001 a
SN-MP (degree)	28.6 (6.3)	29.2 (6.5)	+0.6 (1.3)	0.035 a
FH-AOP (degree)	12.5 (5.1)	10.8 (4.7)	–1.7 (3.1)	< 0.001 a
SN-AOP (degree)	20.0 (5.0)	18.6 (4.4)	–1.4 (2.9)	0.002 a
PP-AOP (degree)	13.7 (4.7)	12.2 (4.9)	–1.5 (3.3)	0.002 a

Abbreviations: ANB, angle between NA plane and NB plane; AOP, anterior occlusal plane; FH, Frankfort horizontal; LAFH, lower anterior facial height; MP, mandibular plane; PP, palatal plane; SN, sella-nasion; SNA, angle between SN plane and NA plane; SNB, angle between SN plane and NB plane.

a Significant.

## Discussion

While each case must be slightly modified to cater to individual patient needs, the deep bite treatment protocol outlined in Table 3 served as the backbone for all treatments evaluated in this study. This deep bite correction protocol is similar to that reported by Kravitz et al as outlined in their work on “Mechanical considerations for deep bite correction with aligners.” 14 In this review, the authors proposed a treatment approach that involved extrusion of the posterior teeth and intrusion of the anterior teeth. They also advocated for the use of bite ramps and class II elastics, particularly in class II or even class I cases, as valuable adjuncts for effectively addressing deep bite cases. In contrast to our protocol, they suggested tilting the lower posterior crowns buccally 5 degrees to achieve “resultant” intrusion of the lower anterior teeth, as necessary. These similarities in protocols suggest clinicians are focusing on establishing standardized protocols capable of accommodating a wide range of deep bite cases. Even though clinicians are attempting to establish standardized protocols, it is important to emphasize that these standardized treatments must be modified to treat the specific needs of each patient. Several factors such as the severity of the deep bite, the vertical skeletal pattern, the origin of the malocclusion, and special biomechanical considerations, all play a role in choosing the appropriate treatment plan for each individual case. 15

The study sample was comprised of class I to mild class II skeletal cases (ANB = 3.2 degrees, SNA = 82.7 degrees, SNB = 79.4 degrees), characterized by a normodivergent with a tendency toward a hypodivergent vertical pattern (SN-MP = 28.7 degrees, LAFH = 61.7 mm). 16 The initial mean value of U1-PP = 107.4 degrees indicated a “normal” relationship of the maxillary incisor to the maxilla. Similarly, the initial mean value of L1-MP = 92.7 indicated a “normal” relationship of the mandibular incisor to the mandible. 17 The anterior overbite started moderately deep (mean of 5.3 mm) with a minimum value of 3.0 mm and a maximum value of 9.7 mm among patients. The sample's overjet initially ranged from a minimum value of 2.3 mm to a maximum value of 7.4 mm resulting in an initial mean of 4.0 mm.

In this study, all patients used bite ramps and 24 out of 37 patients used class II elastics during the course of treatment, both of which contribute to the effectiveness of deep bite correction. 14 For interproximal reduction (IPR), 11 out of 37 patients had a maximum of 0.2 mm of tooth structure removed per contact between the lower incisors. While lower incisors were the most common site of IPR, some patients also received IPR in other regions of the dentition per their specific treatment plans. Data regarding the number of patients that wore class II elastics and had IPR is provided in Table 7 .

**Table 7:** Elastics used and interproximal reduction performed

Class II elastics	Interproximal reduction
24 out of 37 patients	11 out of 37 patients
Average duration of 10.8 months	Approximately 0.2 mm between lower incisors

The Invisalign treatments involved in this study lasted for an average of 21.1 months ( Table 5 ). Prior reports depict groups meeting similar criteria to the class I crowding cases presented herein, but without deep overbites, completed Invisalign therapy in approximately 19.5 months—roughly 1.5 months shorter treatment duration than our group. 18 This difference in treatment duration aligns with the understanding that treating deep bite cases is generally more challenging and more time consuming than addressing minor crowding cases (see Supplementary Material , available in the online version only). 18

Significant changes were observed in 14 of the 19 assessed variables. Notably, most individuals experienced overbite correction of 4.1 ± 1.3 mm and achieved an “ideal overbite value” of 1.1 mm upon completion of treatment. 19 20 These findings suggest that Invisalign can be an effective option for treating moderate dental deep bites. In contrast to the 4+ mm of overbite correction reported herein, a 2017 retrospective study involving a similar age group focusing on deep bite correction, reported only a 1.5-mm decrease in overbite. This correction was primarily attributed to incisor movements, with minimal posterior changes observed. 21 A study from the same year investigated whether the correction of deep bites was influenced by the initial vertical skeletal pattern. This study concluded that lower angle cases tended to correct deep bites primarily through incisor intrusion movements, while higher angle cases relied more on extrusion and uprighting of posterior teeth. 22 Being that our study had a sample of normodivergent, tending toward hyperdivergent patients, one could expect both extrusion of posterior and intrusion of anterior teeth to correct the deep bite. Furthermore, a 2021 study 23 used an “Invisalign (G5) protocol” and virtual bite ramps to correct deep bite malocclusions in adults with a mean initial overbite of 4.5 mm. This study was compared to a control group who were treated with fixed appliances and a mean initial overbite of 4.6 mm. The average age of both groups, 37.2 years, was similar to our study. In terms of overbite correction, the Invisalign group in their study achieved a correction of 1.3 mm while the braces group achieved a 2.0 mm change. Interestingly, our study exhibited a similar vertical change compared to their Invisalign group but achieved better overall overbite correction, including greater lower incisor intrusion and first molar extrusion. Upper incisor intrusion was nearly identical between their study and ours.

Our study included additional variables that may provide further insights into the dental movements that occurred during treatment, such as incisor inclination and vertical movement of the second molars. Two more recent studies attempted to provide insights into the effectiveness of Invisalign and braces in leveling the curve of Spee and the predictability of deep overbite correction using Invisalign. The first study was conducted in 2022 and revealed that Invisalign effectively corrected deep bites, achieving an average correction of 1.6 mm. 24 Notably, this correction appeared to be primarily due to the intrusion of mandibular incisors (with an average intrusion of 2.1 mm), while effectively controlling their proclination. Interestingly, the study also found that, on average, the first and second lower molars intruded by 0.9 and 0.8 mm, respectively, while the upper incisors retroclined on average 2 degrees, resulting in a “relatively” extruded position. It is worth mentioning that this correction was achieved without the use of auxiliary tools, elastics, or precision bite ramps during the treatment phases. In a more recent 2023 study, focused on evaluating the predictability of deep overbite correction using the Invisalign system. 25 Twenty-four individuals with a moderate, initial overbite of 5.2 ± 0.9 mm were treated between 2016 and 2021. The study found that the overbite correction was achieved with an accuracy of 33.4%, resulting in a 1.2-mm improvement out of the planned 3.4 mm correction. The protocol that was used for the virtual set up of the cases included in this study was not clearly mentioned.

The present study demonstrates the efficacy of Invisalign treatment in achieving prescribed extrusion of posterior lower teeth. The results exhibit a systematic and incremental pattern of extrusion across the lower posterior teeth, beginning with the lower second molar and progressing to the first premolar: (L7-MP = +1.0 mm, L6-MP = +1.2 mm, L5-MP = +1.7 mm, L4-MP = +1.7 mm). This progressive extrusion shows the ability of Invisalign treatment in achieving the desired vertical movement of these teeth. These findings hold significant clinical relevance for orthodontic practitioners, offering valuable insights into the latest potential benefits of Invisalign for achieving precise posterior lower teeth extrusion.

The observed transformations in tooth extrusion can be attributed to the advancements introduced in Invisalign's G5 and G8 innovations, particularly designed for the correction of deep bites and our “deep bite protocol.” These enhancements encompass several key elements: (1) *Enhanced control of lower anterior intrusion with lingual surface pressure areas.* G5 and G8 incorporate pressure areas on the lingual surface of aligners, specifically targeting the lower anterior teeth. This strategic placement ensures a more precise application of force along the long axis of these teeth, contributing to controlled and predictable extrusion. (2) *Optimized attachments for bicuspids and molars* . Large and optimized attachments or large horizontal beveled attachments are now employed on all bicuspids and molars. These attachments facilitate more effective activation of extrusive forces on these posterior teeth, enhancing the overall extrusion process. (3) *Precision bite ramps for all upper anterior teeth* . In the majority of cases, precision bite ramps are incorporated on all upper teeth throughout the treatment. These bite ramps serve two essential purposes. They initially aid in the opening of the bite and, equally importantly, help to disclude posterior teeth from any undesired posterior bite forces. This advancement is pivotal in achieving optimal bite correction and it is now automatically placed by the software when certain deep bite criteria are met. (4) *Individualized intrusion for anterior teeth* . The en-masse intrusion of anterior teeth is now executed with forces optimized for each tooth individually. This customized approach ensures that the forces applied align with the unique characteristics and requirements of each tooth, resulting in more precise and effective intrusion. (5) *Leveling the curve of Spee with a smooth transition* . To enhance the overall functionality of the occlusion, G8 introduced improvements in leveling the curve of Spee. In addition, our protocol prescribes a “smooth curve” transition between premolars and anterior teeth instead of a “drastic step” as would most likely be the case in previous years while still preserving a 1.5-mm overcorrection of the deep bite at the virtual case setup. These innovations collectively represent a significant leap forward in the realm of deep bite correction with Invisalign, offering clinicians a more sophisticated and precise treatment approach.

Our lower incisors proclined 4.1 degrees and intruded 1.9 mm improving the deep bite by both true and “relative intrusion” of the lower incisor. This is in agreement with the aforementioned articles about Invisalign's relative effectiveness of deep bite correction through the anterior tooth region. It should be noted that the proclination of the lower incisors is likely due to a combination of relief of lower crowding along with the class II elastic wear by 24 of 37 patients, as mentioned above.

Regarding the upper arch, the upper first molar also extruded by a statistically significant amount of 0.5 mm ± 1.7, though to a lesser extent than the lower molars. Still, this value is nearly double what was measured in a previous study that measured upper molar extrusion. 25 While it was prescribed that both upper and lower posterior teeth be extruded, it was not specified the extent of which to extrude them. It could be that more posterior extrusion was prescribed in the lower than the upper, though this would need to be verified with future studies. For the upper incisors the intrusion of 0.7 mm ± 1.3 was significant and could have aided in the correction of the deep bite as well. This should serve as a caution to clinicians for cases that esthetically would not require upper incisor intrusion to preserve the esthetic incisor display. If it is prescribed, it is likely that some intrusion will occur. Conversely, for those patients with excessive gingival display, slight intrusion of the upper incisor may be a viable option.

With the extrusion of posterior teeth, there is potential for an increase in the vertical dimension. Our study shows that SN-MP increases 0.5 degrees ± 1.4. While this is statistically significant, it shows little clinical significance. LAFH trends toward increasing with a 0.7 mm ± 2.4 increase, though this was not clinically or statistically significant in this study. Still, some caution should be taken on hyperdivergent cases that may be more susceptible to increase in the vertical dimension with posterior teeth extrusion when planning the correction of a deep bite in such cases.

As is expected in a population of nongrowing patients, skeletal values in this study such as SNA, SNB, and ANB did not show statistically significant changes. This shows that the results of treatment did not cause autorotation of the mandible to the degree of changing the patient's skeletal pattern. While some patients did start with a class II dentition, the class II was likely resolved through dental changes using class II elastics.

Regarding the changes in AOP, the AOP decreased as a result of treatment (FH-AOP –1.8 degrees, SN-AOP –1.4 degrees, and –1.5 degrees). This decrease in AOP is likely due to the slight intrusion of the upper incisor. However, the final values of 10.8 and 18.7 degrees for FH-AOP and SN-AOP, respectively, align with those found in class I, pretreatment individuals. 26

Though every precaution was taken, our study still comes with limitations that should be mentioned. While the study showed that Invisalign is effective in correcting a deep bite, further studies should evaluate long-term retention with deep bites and evaluate its stability. While our study had a significant number of cases, a study with more cases would increase confidence in our findings. With a somewhat diverse study group, future studies could isolate more variables such as gender, age, and skeletal classification. Though this study showed that the prescribed tooth movements can be achieved, future studies should evaluate how much tooth movement must be prescribed over the course of treatment in order to achieve these results. Furthermore, patient compliance while wearing removable appliances is difficult to measure, and therefore can cause a misrepresentation in the data if not worn as prescribed. Finally, it can be difficult to locate certain anatomical points using 2D cephalometrics. A 3D analysis of tooth movements relative to cranial structures could give an even more accurate representation of what is occurring when a deep bite is corrected with aligners.

## Conclusion

Invisalign CAT is an effective method to correct deep bites using posterior teeth extrusion and anterior teeth intrusion without significant skeletal opening. While standardized protocols can be useful, each patient must be independently evaluated and each plan must address the patient's specific needs to avoid unwanted side effects. The results presented in this study will help clinicians provide individualized treatment plans through improving our understanding of the dental and skeletal changes that can be expected when correcting deep bites with CAT.
